# Exploration of Factors That Influence Clinical Nurses’ Active Engagement in Research and Evidence-Based Practice: Protocol for a Scoping Review

**DOI:** 10.2196/89824

**Published:** 2026-08-18

**Authors:** Lyndsay Jerusha MacKay, Laurie Lee, Suzanna Crawford, K Alix Hayden, Joshua Yudkin, Sara Moore

**Affiliations:** 1 College of Nursing Texas A&M University Bryan, TX United States; 2 School of Nursing University of Calgary Calgary, AB Canada; 3 Library and Cultural Resources University of Calgary Calgary, AB Canada; 4 School of Public Health Texas A&M University College Station, TX United States; 5 Children's Health Dallas Dallas, TX United States

**Keywords:** nursing research, evidence-based practice, evidence-based nursing, clinical nursing research, professional practice, clinical nursing, research engagement, research use, knowledge translation

## Abstract

**Background:**

Nurses rely on current and relevant evidence to provide safe, high-quality care to patients and families. To do this, nurses need support to participate in research and evidence-based practice (EBP) activities. Despite the well-documented benefits of nurses’ engagement in research and EBP, few actually do so in the clinical setting. This creates a knowledge-to-practice gap in which nursing evidence is not generated and existing evidence is often not applied in practice, jeopardizing the quality of care and patient outcomes.

**Objective:**

The aim of this scoping review is to identify and map the evidence on factors that influence clinical nurses’ engagement in research and/or EBP.

**Methods:**

This review will be conducted in accordance with the JBI methodology for scoping reviews and reported in accordance with the PRISMA-ScR (Preferred Reporting Items for Systematic Reviews and Meta-Analyses Extension for Scoping Reviews) checklist. Searches were conducted on April 28, 2025, in 5 health-related databases. Titles and abstracts, and then full-text articles, were screened by 2 independent researchers against the eligibility criteria for inclusion. The eligibility criteria include research studies (ie, qualitative, quantitative, and mixed methods) and reviews that describe factors that influence nurses working in a clinical setting or in an outpatient clinic attached to a tertiary hospital to engage in research and/or EBP activities. Studies must be published in English after 2010 and conducted in high-income countries. Data will be extracted into a data extraction table adapted from the JBI data extraction instrument by an independent researcher and checked by another. The data from the included studies will be presented in tabular form, accompanied by a narrative summary.

**Results:**

Database searches identified 11,051 records, of which 50.2% (n=5544) remained after duplicate records were removed. Screening began in May 2025, and full-text screening was completed in November 2025. Data extraction and synthesis began in November 2025. Results are planned to be submitted for publication in the summer of 2026.

**Conclusions:**

The findings from this scoping review will inform the development of targeted interventions and organizational strategies that enhance clinical nurses’ engagement in research and EBP. This will better position nurses to improve the quality of care provided to patients and families, thereby improving patient outcomes and advancing the nursing profession.

**Trial Registration:**

Open Science Framework 10.17605/OSF.IO/5VWE9; https://osf.io/5vwe9

**International Registered Report Identifier (IRRID):**

DERR1-10.2196/89824

## Introduction

### Background

Nurses are well positioned to lead the way in health care transformation. They hold the knowledge, skills, and experiences to understand the issues faced by today’s health care systems, promote health, and develop innovative health care models that prioritize well-being and disease prevention [1,2]. As frontline health care providers, nurses have a unique perspective that enables them to identify gaps in care and opportunities to improve the quality of care delivery and the patient experience [3,4]. For nurses to transform health care, they need to be equipped and supported to engage in generating new knowledge through research and integrating high-quality evidence via evidence-based practice (EBP) [2-4]. Nursing research includes systematic inquiry that generates new knowledge and evidence, which can then be evaluated and disseminated to inform nursing practice and advance the profession [3,5]. EBP includes searching, retrieving, appraising, synthesizing, and incorporating current high-quality evidence with patient values and preferences, including context, to improve nursing care [6,7].

It has been well documented that patient safety, quality of care, and cost savings increase dramatically when nurses base their practice on current, relevant, and empirical evidence [6,8]. High-quality nursing care is directly related to positive patient outcomes and satisfaction [9-12]. Despite this, and to the detriment of the patients and populations that nurses serve and care for, very few nurses engage in nursing-specific, clinically oriented research or EBP activities [13]. This has resulted in a knowledge-to-practice gap in nursing practice. When current and relevant evidence exists in the literature, nurses are ill-equipped to access and use this evidence, resulting in the evidence never making it to practice to improve health care [2,14-16]. Additionally, when nurses do not engage in research, generating and creating evidence, there is a gap in the high-quality evidence required to guide their clinical decision-making. Nurses’ engagement in research and EBP activities is the best method for closing the knowledge-to-practice gap and ensuring high-quality patient care [8,17-20].

Clinical nurses spend more one-on-one time with patients and families than any other health care professional. They are exceptionally well-positioned to ask pertinent questions that lead to relevant research projects and EBP activities that improve patient care and outcomes. However, they face significant barriers. Chan et al [21] investigated barriers among 978 emergency nurses engaged in research and found that only 61.8% had completed a research course during their education, and 48.9% reported a lack of knowledge about research study design. Hendricks and Cope [22] conducted a cross-sectional study among 95 nurses to understand barriers to their research use. They found that most nurses have difficulty reading research articles because of research jargon. Other barriers to nurses conducting research and using research findings within their practice include a lack of skills to implement research findings into practice [2,16], an inability to interpret findings from research studies [6], and a perception of the research process as overwhelming [2,16].

Nurses are not supported in conducting research or using evidence to inform their practice. This is of concern because, according to the American Nurses Association [23], the United Kingdom Nursing & Midwifery Council [24], the Nursing and Midwifery Board of Australia [25], and the Canadian Nurses Association [26], nurses must base their practice on the best possible evidence. Therefore, to ensure that nurses can ground their practice in the highest level of evidence and deliver safe, high-quality care, as well as fully realize their roles as health care leaders and drivers of system transformation, health care organizations must implement innovative strategies that foster an environment supportive of nursing research and engagement in EBP. To do this, a comprehensive understanding of the factors that impact nurses’ ability to engage in clinical research and EBP activities is required. Therefore, the aim of this scoping review is to identify and map the evidence on factors that influence clinical nurses’ engagement in research and/or EBP.

### Review Questions

The primary research question for this review is “What factors influence bedside nurses’ engagement in research and EBP activities in the clinical setting?” Subquestions for this review include (1) “What barriers prevent nurses from engaging in research and EBP activities?” (2) “What facilitators enable nurses to engage in research and EBP activities?” (3) “What recommendations have been made to increase the opportunity for nurses to lead research and engage in EBP activities?”

## Methods

### Study Design

This scoping review will be conducted in accordance with the JBI methodology for scoping reviews [27] and reported in accordance with the PRISMA-ScR (Preferred Reporting Items for Systematic Reviews and Meta-Analyses Extension for Scoping Reviews) checklist (Multimedia Appendix 1) [28]. It has been registered prior to project initiation with the Open Science Framework [29].

### Search Strategy

Following the JBI (2020) guidelines, a 3-step process was used to develop the final search strategy. First, an initial search was conducted in Google Scholar to identify seed studies that met all the inclusion criteria and triggered none of the exclusion criteria. Next, the relevant keywords related to “engagement” were identified in the titles and abstracts of the seed studies. These keywords, along with keywords associated with EBP or research, were used to develop a search strategy in the MEDLINE ALL (Ovid) database. In addition, the search strategies from the reviews conducted by Boaz et al [30] and Chalmers et al [31] were adapted and incorporated into the final search strategy for this review. Prior to database translation, the final MEDLINE search was pilot-tested with the seed studies to ensure that all were captured by the search.

The final search used proximity searching to focus results on nurse engagement with EBP or research. Keywords for barriers or facilitators were not incorporated to ensure that the search was comprehensive and that studies would not be missed.

Databases searched included MEDLINE ALL (Ovid), Embase (Ovid), APA PsycINFO (Ovid), CINAHL Plus with Full Text (EBSCOhost), and Scopus. The search was the same for all databases, except for the proximity operator, which was translated to be appropriate for each database platform. The search was not limited by date or language. The final searches for all databases are available in Multimedia Appendix 2.

### Eligibility Criteria

The inclusion and exclusion criteria are reported using the population, concept, and context (PCC) framework and article type as per JBI [27].

#### Population

Registered nurses, nurse practitioners, and advanced practice registered nurses who work in the clinical setting. As it can be challenging to identify the level of training and education of nurses reported in studies, we also included licensed practical nurses; however, nursing attendants and nursing aides were excluded. Roles of nurses within the clinical setting included bedside or clinical nurses, advanced practice nurses, administrators, managers, nurse researchers, and clinical nurse specialists. Nurses who were actively enrolled in graduate studies were included; however, student nurses who were enrolled in an undergraduate program were excluded. Additionally, studies that included physicians and allied health care professionals were excluded, unless 50% of the sample consisted of nurses.

#### Concept

The core concept captured in this review is the factors that influence nurses’ ability to engage in research and EBP activities. For this review, 2 conceptually distinct but related constructs are operationalized as separate analytic terms. The first is research engagement, which is defined as systematic inquiry that generates new knowledge and evidence, which can then be evaluated and disseminated to inform nursing practice and advance the nursing profession [3,32]. The second is EBP engagement, which is defined as searching, retrieving, appraising, synthesizing, and incorporating current high-quality evidence with patient values, preferences, and context into the nursing care provided to patients and families [6]. Both constructs share the overarching goal of closing the knowledge-to-practice gap; however, they are treated as two different concepts.

Articles that discussed and described nurses working on clinical trials that are not nursing-led studies were excluded. Articles that discussed nurses’ engagement in research and EBP during the COVID-19 pandemic were excluded because of the extraordinary and acute demands of the pandemic era that are not typical of clinical nursing environments (ie, redeployment, staffing crises, and suspension of nonurgent research and EBP activities). This represents conditions that are not typical of organizational- and individual-level factors and therefore are unlikely to be transferable to the design of strategies and approaches to increase nurses’ engagement in research and EBP during noncrisis interventions.

#### Context

Inpatient care units included intensive care units, emergency departments, oncology units, mental health units, neonatal intensive care units, and outpatient care units. Outpatient care clinics were included, provided that they were attached to or associated with a tertiary care hospital. Excluded settings were community health offices, physician’s offices (family physicians), rural health, public health, and academia. Articles reporting research studies were limited to those conducted in high-income countries, as defined by the United Nations Department of Economic and Social Affairs in the World Economic Situation and Prospects report [33]. Multimedia Appendix 3 lists the 37 countries. This was done because our objective is to inform strategies that can be implemented within North American health care systems, and health care systems within the 37 included countries have similar resource levels, workforce infrastructure, regulatory expectations for nurses, and organizational infrastructure that supports research and EBP. Including studies from low- and middle-income countries, where workforce structures, educational preparation for nurses, and institutional research support differ substantially, would risk conflating structural barriers and facilitators. Therefore, such countries were excluded to ensure that the findings result in actionable recommendations specific to comparable health care contexts. Review articles conducted by authors from any country were included.

#### Types of Sources

Qualitative (eg, phenomenology, grounded theory, ethnography, and thematic analysis); quantitative (randomized controlled trials, quasi-experimental, and observational); and mixed methods research studies were included. Reviews (eg, systematic reviews, meta-analyses, literature reviews, and scoping reviews); project descriptions; quality improvement studies; discussion papers; and case studies published in English in 2010 or later were also included. A time limit was included in the eligibility criteria after the search was conducted because there was an extremely large number of included studies, and studies published before 2010 were excluded to make the number of included studies more manageable. Additionally, this time limit ensures that the evidence reflects contemporary models of health care delivery, the current conceptualization of EBP, and modern expectations for research engagement in nursing practice. A gray literature search, including government reports, dissertations, theses, editorials, opinion pieces, and conference abstracts, as well as backward citation searching of included articles, was not conducted because of the large number of included studies identified through database searches, which provided sufficient breadth of evidence and made these additional steps infeasible within the scope of this review.

### Study Selection

All titles and abstracts from the database searches were uploaded into Covidence (Veritas Health Innovation) [34], and duplicate records were removed. Covidence is useful for managing and screening large numbers of titles and abstracts and PDF files of research articles, facilitating blinding, and assisting with conflict resolution. Reviewers pilot-tested the inclusion and exclusion criteria on 50 randomly selected titles and abstracts until they reached a minimum interrater reliability of 90%, which required 2 rounds. Screening occurred in 2 phases. In phase 1, all titles and abstracts were screened against the inclusion and exclusion criteria by 2 independent reviewers in Covidence. In phase 2, the titles and abstracts included from phase 1 were retrieved in full text. The full-text articles were read in full and screened against the inclusion and exclusion criteria by 2 independent reviewers. Any disagreements at either phase were resolved via discussion between the two reviewers. Reasons for exclusion were documented. The results from the search and screening process will be reported in a PRISMA-ScR flow diagram [27,28]. Because of the large number of included studies, the bibliographic references of included articles will not be examined to identify additional studies.

### Data Extraction

Data from the included articles will be extracted by 1 reviewer and checked by another reviewer into a data extraction table adapted from the JBI data extraction instrument (Multimedia Appendix 4) [27]. This approach was selected in lieu of dual independent extraction given the large volume of included studies and the descriptive, nonevaluative nature of the scoping review data extraction. The data extraction table will be pilot-tested on the first 10 articles that appear in alphabetical order to ensure that all important study information is included. Elements of the data extraction table include authors; date; country; aim, purpose, and research questions; sample with participant characteristics; methods or study design; intervention (if used); measurement tools or theoretical framework (if used) or outcome measures; barriers; facilitators; overall findings; and recommendations. The data extraction instrument will be adjusted and adapted as required during the data extraction process. In line with JBI recommendations, critical appraisal is not mandatory for scoping reviews unless the review aims to inform practice recommendations [35].

### Data Analysis and Presentation

The extracted data will be descriptively mapped and presented in tabular form using tables and figures. First, a table reporting the study information from each article will be developed to report the author, date, country, aim or research question, study method or design, sample characteristics, intervention (if used), theoretical framework (if used), barriers, facilitators, overall findings, and recommendations. Second, a table will be created to report the study characteristics using frequency counts of the following: country, date of publication, study method or design, participant characteristics, theoretical framework (if used), and intervention (if used). These tables will be supplemented with a narrative synthesis to provide a detailed description of the factors influencing nurse engagement in clinical research and EBP activities. This will be accomplished by conducting a thematic analysis [36] of the data in two parallel analytic streams: (1) research engagement (barriers, facilitators, and recommendations specific to factors influencing nurses’ engagement in knowledge generation activities) and (2) EBP engagement (barriers, facilitators, and recommendations specific to factors influencing nurses’ engagement in knowledge use). If data from included articles are reported in a manner in which this clear distinction cannot be made, because research and EBP are sometimes grouped or the terms can be used interchangeably, those articles will be grouped and the data will be analyzed separately. For this thematic analysis, first, data will be coded; then, codes will be grouped into categories according to recurring patterns among the codes; and finally, categories will be collapsed into themes. This process is intended to categorize and descriptively synthesize the evidence rather than produce a qualitative interpretation.

### Ethical Considerations

This scoping review protocol does not involve the collection of data from human subjects; therefore, ethics approval from a research ethics committee is not required.

## Results

A total of 11,051 records were identified through the database searches, of which 50.2% (5544/11,051) records remained after duplicate records were removed. Screening began in May 2025, and full-text screening was completed in November 2025, resulting in the inclusion of 5.3% (293/5544) articles. Data extraction and synthesis began in November 2025 and are planned to be completed by the spring of 2026, with publication of the final results in the summer of 2026.

## Discussion

### Anticipated Findings

It is anticipated that the findings from this review will summarize and present the factors that influence clinical nurses’ engagement in research and EBP activities. Specifically, the findings are expected to reveal multiple categories of barriers that prevent nurses’ engagement and multiple categories of facilitators that enable nurses’ engagement. It is also anticipated that organizational, leadership, and individual-level factors that influence nurses’ ability to engage in research and EBP will be highlighted.

### Comparison With Prior Work

Existing reviews have examined aspects of nurses’ research and EBP engagement, but none have provided an overview across both research and EBP for clinical nurses in high-income countries. A preliminary search was conducted in January 2025 via Google Scholar and the MEDLINE database to confirm that no current or existing scoping reviews or systematic reviews comparable with this scoping review had been conducted previously. However, there are related reviews. For example, Morrison et al [37] conducted a mixed methods systematic review to investigate factors that influence nursing research activities among clinical nurses. However, they only included 16 studies that focused primarily on research, and their results section was presented as a description of the studies rather than as a narrative summary and synthesis of the data. Squires et al [38] conducted a systematic review to identify the extent to which nurses use research findings in practice. This review is approximately 15 years old and does not include nursing-led research activities. Furtado et al [39] conducted a scoping review on environmental and organizational factors that affect nurses’ adoption of EBP. This review included students, whom we excluded, and only focused on EBP and organizational and practice environmental factors. The findings from this scoping review will present a comprehensive and complete picture of the factors that influence nurses’ engagement in both research and EBP activities, complementing the findings of these published reviews while adding additional insight and understanding.

### Strengths and Limitations

The current scoping review rigorously follows the JBI methodology for scoping reviews. Both screening phases used 2 independent reviewers to minimize selection bias. Pilot testing of the inclusion and exclusion criteria was completed on 50 titles and abstracts until 90% interrater reliability was reached. Data extraction will be performed by 1 reviewer and verified by a second. The inclusion criteria are broad in representation, which could result in the included studies being global in nature, including all high-income countries across various clinical and outpatient settings. Therefore, the findings may be applicable to a variety of settings and provide an understanding of the practical application of factors that facilitate nurse engagement in research and EBP across a variety of organizations and health care systems.

The included articles are limited to those published in English; therefore, relevant articles may be missing. The countries were limited to only high-income countries, which limits the global perspective and application to low- and middle-income countries. This reduces transferability to geographic areas in low-income countries. Gray literature was not searched; therefore, relevant government reports, dissertations, and conference proceedings may be missing. Finally, quality appraisal will not be completed; hence, the effectiveness of interventions cannot be addressed.

### Future Directions and Implications

The thematic analysis of barriers, facilitators, and recommendations is expected to identify strategies that leaders within health care organizations can use to design and implement multilevel strategies to enhance clinical nurses’ engagement in research and EBP. These findings can inform the development of future studies that test the recommended strategies identified in the findings. Finally, the information included in the findings can be used to guide clinical education curricula and inform undergraduate nursing education.

### Dissemination Plan

The findings of this scoping review will be disseminated through peer-reviewed journal publications, presentations at nursing and implementation science conferences, and tailored summaries for clinical nurses, nursing leaders, academic schools of nursing, and professional organizations.

### Conclusions

Nurses must incorporate current evidence into their practice to ensure that the care they provide to patients and families is safe and high-quality, promoting positive outcomes. To do this, nurses need to be supported in conducting research that generates new evidence and be empowered to identify and implement evidence in their practice. This scoping review protocol outlines a comprehensive approach to systematically identify and map the evidence on factors that influence clinical nurses’ engagement in research and EBP activities. The findings from this scoping review will offer clinical nurses, nurse researchers, academics, hospital administrators, and health care leaders an in-depth understanding of what hinders nurses’ engagement in research and EBP, along with established strategies that they can use to develop and implement interventions and programs for improvement.

## Appendix

Multimedia Appendix 1 PRISMA (Preferred Reporting Items for Systematic Reviews and Meta-Analyses) checklist.

Multimedia Appendix 2 Final searches for all databases.

Multimedia Appendix 3 List of 37 developed nations as per the United Nations Department of Economic and Social Affairs.

Multimedia Appendix 4 Data extraction template.

## Abbreviations

EBP: evidence-based practice
PCC: population, concept, and context
PRISMA-ScR: Preferred Reporting Items for Systematic Reviews and Meta-Analyses Extension for Scoping Reviews
