# Supplementary material for: Exploration of Factors That Influence Clinical Nurses’ Active Engagement in Research and Evidence-Based Practice: Protocol for a Scoping Review
Source: JMIR Res Protoc. 2026 Aug 18;15:e89824. doi: 10.2196/89824 (PMC13535305; doi:10.2196/89824)
Supplement: Supplementary file 3 [file resprot_v15i1e89824_app3.docx]

**Supplementary File 3**

**37 Developed Nations as per the United Nations Department of Economic and Social Affairs ‘World Economic Situation and Prospect Report’ (2025)**

United States

Canada

Japan

Republic of Korea

Australia

New Zealand

Austria

Belgium

Bulgaria

Croatia

Cyprus

Czechia

Denmark

Estonia

Finland

Germany

Greece

Hungary

Ireland

Italy

Latvia

Lithuania

Luxembourg

Malta

Netherlands

Polan

Portugal

Romania

Slovakia

Slovenia

Spain

Sweden

Iceland

Norway

Switzerland

United Kingdom
