# Supplementary material for: Exploration of Factors That Influence Clinical Nurses’ Active Engagement in Research and Evidence-Based Practice: Protocol for a Scoping Review
Source: JMIR Res Protoc. 2026 Aug 18;15:e89824. doi: 10.2196/89824 (PMC13535305; doi:10.2196/89824)
Supplement: Supplementary file 4 [file resprot_v15i1e89824_app4.docx]

| Author, Date, Country | Aim/Purpose/Research Question(s) | Sample with Participant Characteristics | Method/Study Design | Intervention | Measurement tools/Theoretical framework/Outcome measures | Main Results: Barriers | Main Results: Facilitators | Overall Findings/Recommendations/Outcomes |
| --- | --- | --- | --- | --- | --- | --- | --- | --- |
|  |  |  |  |  |  |  |  |  |

**Supplementary File 4: Data Extraction Template**
